# Content analysis of Netflix and Amazon Prime Instant Video original films in the UK for alcohol, tobacco and junk food imagery

**DOI:** 10.1093/pubmed/fdab022

**Published:** 2021-03-02

**Authors:** Khaldoon Alfayad, Rachael L Murray, John Britton, Alexander B Barker

**Affiliations:** UK Centre for Tobacco and Alcohol Studies, Division of Epidemiology and Public Health, Faculty of Medicine, University of Nottingham, Clinical Sciences Building, City Hospital, Nottingham NG5 1PB, UK; Department of Public Health, College of Health Sciences, Saudi Electronic University, Riyadh Campus, Riyadh 13323, Saudi Arabia; UK Centre for Tobacco and Alcohol Studies, Division of Epidemiology and Public Health, Faculty of Medicine, University of Nottingham, Clinical Sciences Building, City Hospital, Nottingham NG5 1PB, UK; SPECTRUM Consortium, UK; UK Centre for Tobacco and Alcohol Studies, Division of Epidemiology and Public Health, Faculty of Medicine, University of Nottingham, Clinical Sciences Building, City Hospital, Nottingham NG5 1PB, UK; SPECTRUM Consortium, UK; UK Centre for Tobacco and Alcohol Studies, Division of Epidemiology and Public Health, Faculty of Medicine, University of Nottingham, Clinical Sciences Building, City Hospital, Nottingham NG5 1PB, UK; SPECTRUM Consortium, UK

## Abstract

**Background:**

Exposure to alcohol, tobacco and high fat, sugar and salt (HFSS) food imagery is a significant risk factor for the uptake and regular use of these products in young people, and imagery are more frequently portrayed in video-on-demand (VOD) than in terrestrial television programmes. This study compared alcohol, tobacco and HFSS imagery in original films on Amazon Prime Instant Video and Netflix.

**Methods:**

Content analysis of 11 original films released by Amazon Prime and Netflix in 2017 using 5-minute interval coding of alcohol, tobacco and HFSS content. Proportions of intervals containing alcohol, tobacco and HFSS imagery were compared between services using the chi-square test.

**Results:**

Alcohol content appeared in 200 (41.7%) out of the total of 479 intervals coded, whereas tobacco and HFSS appeared in 129 (26.9%) and 169 (35.24%), respectively. Proportions were similar between Amazon Prime Instant Video and Netflix original films and were unrelated to film age classification.

**Conclusions:**

Alcohol, tobacco and HFSS content likely to promote consumption among young people occurs frequently in original films shown by VOD services in the UK. Further studies are needed to investigate effective regulatory frameworks for VOD services to protect viewers from harmful or unwanted contents.

## Introduction

Exposure to tobacco, alcohol and high fat, sugar and salt (HFSS) food imagery in the media is associated with increased use and consumption of these products among young people,^1–6^ hence contributing to significant current and future disease and cost burdens on society.^7–10^ However, while tobacco and alcohol content in UK broadcast media is now increasingly well documented, relatively little is known about HFSS imagery in popular programming. This is particularly true of the content of video-on-demand (VOD) services such as Netflix and Amazon Prime Instant Video, which are becoming increasingly popular when compared with terrestrial television services.^11^ Globally, Netflix has 158 million subscribers and ~11.8 million subscribers in the UK alone,^12^^,^^13^ whereas Amazon Prime Instant Video has 150 million and 8 million subscribers worldwide and in the UK, respectively.^14^^,^^15^^,^^31^ These services are particularly popular among young people, with 20% of young adults (16–24 years) and 46% of teenagers in the UK having used VOD services such as Netflix.^14^

Online VOD services are subject to content regulations applied in the country in which they are registered. Amazon Prime Instant Video is registered in the UK and is regulated by the Office of Communications (Ofcom),^16^ which prohibits the inclusion of smoking and alcohol consumption in programmes made primarily for children and recommends that these activities are not condoned, encouraged or glamorized in programmes that are likely to be seen by people <18 years old.^16^ Ofcom’s regulation also prohibits the advertising of HFSS in programmes made primarily for children but does not cover HFSS content in programmes, which are not primarily made for children but can be seen by children.^33^ In contrast, Netflix is registered in Europe and subject to regulation by the European Regulators Group for Audio–Visual Media Regulators, which applies controls on content deemed harmful to young people but does not comment on non-commercial portrayals of alcohol, tobacco or HFSS use in programme content such as programmes that could seriously impair the mental or moral development of minors.^17^^,^^29^

Our own and others’ work has demonstrated that tobacco and alcohol imagery is portrayed more frequently in VOD than terrestrial television programming.^14^^,^^29^ However, no study to date has investigated alcohol, tobacco and HFSS imagery in original films made for and distributed by VOD services. We have therefore provided descriptions of alcohol, tobacco and HFSS imagery between providers and in relation to age rating, in selected films on Amazon Prime Instant Video and Netflix.

## Methods

Amazon Prime released 11 original films (excluding documentaries) in 2017, all of which are included in this study. Netflix released a large number of films in 2017, and we sampled the 11 films with the highest ranking on the Internet Movie Database (IMDb).^18^

We recorded the British Board of Film Classification (BBFC)^19^ classification and genre for each of the selected films and coded content using an adaptation of a 5-minute interval coding method used extensively in the past to code tobacco and alcohol content.^14^^,^^20^^,^^21^

In each 5-minute interval, we recorded, separately for alcohol, tobacco and HFSS imagery, content in the following categories:

- Actual consumption/use: the actual consumption/use of alcohol, tobacco and/or HFSS food by any character.
- Inferred consumption/use: reference to the consumption/use of alcohol, tobacco and/or HFSS food without actual consumption/use. This includes verbal or non-verbal reference to alcohol, tobacco and/or HFSS, inferred alcohol consumption and alcoholic behaviour. Actions not included in this list that suggest the consumption of these items are categorized as others.
- Paraphernalia/other reference: the appearance or presence of materials related to alcohol, tobacco and/or HFSS without actual or implied consumption/use.
- Branding: the presence of materials associated to alcohol, tobacco and/or HFSS.

We categorized alcohol types as beer, wine/champagne and spirits; used a ‘various types’ category to describe types when more than one was displayed simultaneously and an ‘unknown’ category to capture the occurrence of other forms of alcohol (different from beer, wines/champagne or spirits). Inferred alcohol use was categorized as inferred consumption (such as when an actor holds a glass appearing to contain an alcoholic drink), verbal reference to alcohol consumption or the display of alcoholic behaviour (such as slurred speech, loss of balance while walking and lack of coordination). The appearance of alcohol bottles, verbal conversation including the word alcohol and the appearance of other items (such as beer can, drinking glasses) were categorized as alcohol paraphernalia. The presence of alcohol brands whether consumed or not were categorized as alcohol brands.

For tobacco, we coded actual use as the smoking of any of three types of tobacco product: cigarettes, cigars and pipes. Inferred us comprised verbal and non-verbal (such as holding a cigarette, cigar or smoking pipe) references to tobacco smoking. The display of smoking-related items such as ashtrays, cigarette butts, cigarettes, lighters, matches and smoking/non-smoking signs) were categorized as paraphernalia/other reference, whereas the display of any related materials to tobacco that are not listed were categorized as a subcategory of paraphernalia/reference materials named others. The presence of tobacco brands whether they were smoked or not were categorized as tobacco brands.

Food items were identified as being HFSS foods by their likely inclusion into the Ofcom ‘Big 5’ categories of HFSS food; branded foods were checked to make sure that they are HFSS foods using the UK Standard Agency nutrient profiling model^32^. HFSS content were categorized as either actual consumption, implied consumption, paraphernalia or branding. With respect to HFSS, the consumption of food items containing high fat (such as fast food, burgers, chips and pre-prepared convenience foods), high sugar (such as soft drinks, confectionaries and pre-sugared cereals) and high salt (such as crisps) was considered as actual consumption.

Implied HFSS consumption were categorized similarly to the categorization for tobacco: verbal, non-verbal (such as actors holding soft drinks candy bars or other HFSS without actually consuming it) or other gestures suggesting the consumption of HFSS were categorized as inferred use. The actual display of food or food packets, food advertising, conversation about HFSS (verbal, not implied) as well as the display of HFSS-associated branding or any other sign that suggests HFSS consumption were considered HFSS paraphernalia. The presence of HFSS brands whether they were consumed or not were categorized as HFSS brands.

Multiple appearances of the same category within the same 5-minute interval were recorded as one event unless the appearance overlapped two (or more) intervals, in which case it was recorded as two (or more) events. The proportion of viewing time showing each type of imagery was computed by dividing the number of all intervals showing the imagery by the total number of intervals coded. Coding data were initially recorded in Microsoft Excel prior to analysis using IBM SPSS statistical package (version 24). The proportion of 5-minute intervals containing alcohol, tobacco or HFSS imagery were compared between the two VOD services using the chi-square test to explore any possible differences between them to explore whether the differences in regulation result in differences in the amount of content shown.

## Results

The 22 films provided a total of 2357 minutes of run time, which we coded in 479 five-minute intervals (235 from Netflix and 244 from Amazon Prime Instant Video content). The names, genres and BBFC age ratings of the films are listed in Table 1. All but one of the films was rated as suitable for viewing by persons aged under 18.

**Table 1:** Summary of IMDb classification and ranking, and BBFC rating of selected films

***Source***	***Movie title***	*^**ǂ**^ **IMDb classification***	*^**∆**^ **BBFC rating***	*^**ǂ**^ **IMDb ranking***
Amazon Prime Instant Video	The Salesman	Drama	12A	7.8
	The Lost City Z	Adventure	15	6.6
	The Wall	Drama	15	6.2
	The Big Sick	Comedy	15	7.5
	Landline	Comedy	(R)	6.4
	The Only Living Boy in New York	Drama	(R)	6.3
	Crown Heights	Drama	(R)	6.5
	Brad’s Status	Comedy	15	6.5
	Wonderstruck	Drama	PG	6.2
	Last Flag Flying	Comedy	15	6.9
	Wonder wheel	Drama	12A	6.2
Netflix	Okja	Action	15	7.3
	Imperial Dreams	Drama	15	6.7
	I Do not Feel at Home in This World Anymore	Comedy	15	6.9
	Tramps	Comedy	15	6.5
	First They Killed My Father	Drama	15	7.2
	Gerald’s Game	Drama	18	6.6
	Bright	Action	15	6.3
	Our Soul at Night	Drama	PG	6.9
	To The Bone	Drama	15	6.8
	The Incredible Jessica James	Comedy	15	6.5
	The Meyerowitz Stories	Comedy	15	6.9

^**ǂ**^According to the IMDB (https://www.imdb.com)

^**∆**^According to the BBFC (http://www.bbfc.co.uk/)

**Fig. 1:**
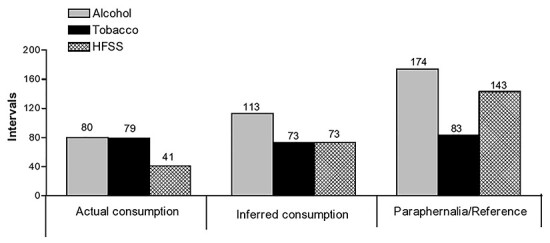
Summary of data on actual consumption, inferred consumption and reference to alcohol, tobacco and HFSS in all films selected for analysis.

In total, alcohol content was seen in 200 (41.7%) of the 479 intervals of film coded and included 80 intervals of actual alcohol consumption, 113 intervals of inferred use and 174 intervals of alcohol paraphernalia/other reference content. Tobacco appeared in 129 (26.9%) intervals, with actual tobacco use, inferred tobacco consumption and tobacco paraphernalia/other reference, respectively, occurring in 79 (16.4%), 73 (15.2%) and 83 (17.3%) intervals. HFSS content appeared in 169 (35.2%) intervals, with actual consumption of HFSS occurring in 41 (8.5%), inferred consumption in 73 (15.2%) and paraphernalia/other reference to HFSS consumption in 143 (29.8%) intervals (Fig. 1).

### Alcohol imagery

For actual consumption, most of the alcohol imagery related to beer (37 intervals, 46.3% of actual consumption intervals), followed by wine/champagne (21 intervals, 26.3%), with only one interval of spirit consumption. Multiple types of alcohol product were consumed in seven (8.8%) intervals (Table 2). There were 94 intervals (19.6% of the total intervals) of inferred alcohol, of which 26 intervals (27.7% of inferred intervals) of verbal inferred alcohol, and this is twice the occurrence observed for alcoholic behaviour (13 intervals, 13.8% of inferred intervals), whereas only one other types of inferred alcohol use was observed in the study (Table 2). The appearance of bottles was the most common alcohol reference observed (108 intervals, 22.5% of the total intervals). Moreover, Table 2 showed that other forms of reference to alcohol consumption occurred in 81 intervals (75% of other content intervals), whereas verbal reference to alcohol consumption was observed in 41 intervals (37.9% of other content intervals). Furthermore, there were 29 intervals of alcohol brands (6.1% of total intervals).

**Table 2:** Details of data on imagery of alcohol, tobacco and HFSS from all selected films

***Coding category***	***Product category***	***Brand/activities/materials***	***Interval***			***Percentage (%)***
			***Amazon***	***Netflix***	***Total***	
Actual use	Alcohol	Beer	15	22	37	7.7
		Wine/Champagne	14	7	21	4.3
		Spirits	1	0	1	0.2
		Various types	7	0	7	1.5
		Unknown	16	6	22	4.5
		**Total**	**53**	**35**	**88**	**18.3**
	Tobacco	Cigarette	42	29	71	14.8
		Cigar	8	2	10	2.1
		Smoking pipes	1	2	3	0.6
		**Total**	**51**	**33**	**84**	**17.5**
	HFSS	Soft drink	12	3	15	3.1
		Confectionaries	5	4	9	1.8
		Pre-sugared breakfast cereal	2	0	2	0.4
		Crisps and savoury snacks	4	0	4	0.8
		Fast food	5	3	8	1.6
		Pre-prepared convenience food	4	3	7	1.5
		**Total**	**32**	**13**	**45**	**9.4**
Inferred use	Alcohol	Inferred drinking	56	38	94	19.6
		Verbal	16	10	26	5.4
		Alcoholic behaviour	7	6	13	2.7
		Others	1	0	1	0.2
		**Total**	**80**	**54**	**134**	**27.9**
	Tobacco	Verbal	3	2	5	1
		Non-verbal	40	29	69	14.4
		**Total**	**43**	**31**	**74**	**15.4**
	HFSS	Verbal	8	9	17	3.5
		Non-verbal	40	27	67	13.9
		Others	1	0	1	0.2
		**Total**	**49**	**36**	**85**	**17.7**
Paraphernalia /Reference	Alcohol	Bottles	54	54	108	22.5
		Verbal reference	27	14	41	8.5
		Others	53	28	81	16.9
		**Total**	**134**	**96**	**230**	**48**
	Tobacco	Ashtray	29	20	49	10.2
		Cigarette display	20	13	33	6.8
		Lighter	10	12	22	4.5
		Matches	6	1	7	1.5
		Smoking/non-smoking sign	10	3	13	2.7
		Others	9	0	9	1.8
		**Total**	**75**	**49**	**124**	**25.8**
	HFSS	Display of food packet	31	32	63	13.1
		Advertising	11	8	19	3.9
		Actual food shown	26	24	50	10.4
		Verbal, not implied	20	19	39	8.1
		Others	7	3	10	1.5
		**Total**	**95**	**86**	**182**	**37**
Brands	Alcohol	Brands	21	8	29	6.1
	Tobacco	Brands	1	0	1	0.2
	HFSS	Brands	24	35	59	12.3

### Tobacco imagery

In total, tobacco content was seen in 129 intervals (26.9% of total intervals). Tobacco use was seen in 79 intervals, whereas cigarettes were the most frequently consumed tobacco product (71 intervals, 89.8% of tobacco use intervals) with use of cigars and pipes occurring in 10 (12.7%) and 3 (3.8%) intervals, respectively (Table 2). Implied use occurred in 74 intervals of which non-verbal use was observed in 69 intervals (93.2% of implied use intervals), whereas verbal implied tobacco use was observed only in five intervals (6.8% of implied use intervals). Tobacco paraphernalia/other reference was recorded in 92 intervals, comprising the display of ashtrays (49 intervals, 53.3 of paraphernalia intervals), cigarettes (33 intervals, 35.9% of paraphernalia intervals), lighters (22 intervals, 23.9% of paraphernalia intervals), matches (seven intervals, 7.61% of paraphernalia intervals) and of smoking or ‘no smoking’ signage in four intervals. Other forms of reference to tobacco smoking were observed in nine (9.57%) intervals. Tobacco brand was seen in one (0.2% of the total intervals) interval (Table 2).

### HFSS imagery

In total, HFSS content was seen in 169 intervals (35.3% of total intervals). HFSS consumption was seen in 41 intervals (8.5% of the total intervals); soft drinks (15 intervals, 36.6% of consumption intervals) were the most prevalent type of HFSS consumed, followed by confectionaries (nine intervals, 22% of consumption intervals). Fast food (eight intervals, 19.5%), pre-sugared breakfast cereal (two intervals, 4.9%), crisps and savoury snacks (four intervals, 9.8%) and pre-prepared convenience food (seven intervals, 17.1%) were also observed. Inferred consumption was seen in 73 intervals (15.2% of the total intervals). Non-verbal and verbal inferred HFSS consumption was observed in 67 intervals (91.8% of inferred consumption intervals) and 17 intervals, respectively (23.3% of inferred consumption intervals). Paraphernalia/other reference to HFSS consumption was seen in 143 (29.8%) intervals. Display of food packets (63 intervals, 44% of paraphernalia intervals) and actual food (50 intervals, 35% of paraphernalia intervals) were the most common HFSS paraphernalia/reference observed (Table 2). Verbal but not implied references (39 intervals, 27.3% of paraphernalia intervals), HFSS advertising (19 intervals, 13.3% of paraphernalia intervals) and other forms of HFSS reference (1 interval) were also observed. HFSS brand (59 intervals, 12.3%).

### Comparison of alcohol, tobacco and HFSS imagery in Amazon Prime Instant Video and Netflix films

The total number of intervals showing alcohol, tobacco and HFSS imagery in Netflix films was 433 compared with 613 intervals observed for Amazon Prime Instant Videos. There was no significant difference in alcohol, tobacco and HFSS imagery observed in films selected from Amazon Prime Instant Video compared with Netflix (Table 2). A comparison of the proportion of intervals showing alcohol, tobacco and HFSS in films selected from Amazon Prime Instant Video and Netflix is presented in Table 3. While the total proportion of alcohol and HFSS intervals were the same on each service, a slightly higher proportion of tobacco content intervals was observed on Amazon Prime Instant Video. Overall comparison of intervals for alcohol, tobacco and HFSS imagery across all the categories indicates that there was no significant difference between the both services.

**Table 3:** Proportion of intervals containing content on each service

*Product*	*Data Category*	*Netflix*	*Amazon Prime Instant Video*	P
Alcohol	Any Content	42	42	1
	Use	13	20	0.253
	Inferred Use	18	28	0.130
	Paraphernalia	36	36	1
	Branding	9	3	0.134
Tobacco	Any Content	24	30	0.426
	Use	13	20	0.253
	Inferred Use	14	18	0.563
	Paraphernalia	16	20	0.581
	Branding	1	0	1
HFSS	Any Content	35	35	1
	Use	5	12	0.126
	Inferred Use	12	18	0.322
	Paraphernalia	31	28	0.757
	Branding	10	14	0.515

## Discussion

### Main finding of this study

The current study found no significant difference in the proportion of intervals containing alcohol, tobacco and HFSS imagery in original Netflix films and original Amazon Prime films, showing that the stricter Ofcom regulations applied to Amazon Prime do not necessarily result in less content being shown. Our findings indicate that original films on VOD services are likely to be a source of harmful exposure to tobacco, alcohol and HFSS imagery in young people. Promoting stricter regulations has to be considered by governments and the regulatory parties as with current regulations are failing to stop or reduce the high content of tobacco, alcohol and HFSS in VOD services. In view of the changing viewing habits of adolescents, there is a need for the regulation of tobacco, alcohol and HFSS imagery in original content from VOD services, whether this be updating the European Audio–Visual Media Services directive to ensure that European regulations specifically include alcohol, tobacco and HFSS content or ensuring that the current UK regulations that cover Amazon Prime Instant Video are applied. Regulating this content on VOD services would likely prevent youth exposure to this content.

### What is already known in this topic

This study is the first content analysis of HFSS imagery in original films made by and distributed by VOD services. This study adopted semi-quantitative 5-minute interval coding methods similarly established in previous studies to the analysis of alcohol and tobacco contents of both terrestrial television programmes and programmes provided via VOD services.^14^^,^^24–26^ Findings of the present study confirm previous reports indicating potential harmful exposure to alcohol and tobacco imagery in programmes provided by VOD services,^14^ with Amazon Prime films having higher number of imageries compared with Netflix films. We selected Netflix films for analysis using IMDB ratings as opposed to the selection of top films per year by viewership as we have done in our previous studies,^24–26^ since viewing data for VOD programmes are not available.

### What this study adds

This study demonstrates that films originally made for and distributed by Amazon Prime Instant Video and Netflix contain a considerable amount of alcohol, tobacco and HFSS imagery. Our study has also revealed that the frequency of display and the proportion of programming time containing these imageries were not significantly different in original films selected from Amazon Prime Instant Video compared with films selected from Netflix. This implies that these services equally represent a source of exposure to alcohol, tobacco and HFSS content to viewers, despite the differences in regulations applying to these two providers and the generally stricter controls applying to Amazon Prime Instant Video.^22^^,^^23^ This indicates that the stricter regulation imposed by Ofcom may not necessarily translate to proportional reduction in alcohol, tobacco and HFSS imagery in programmes.

### Limitation of this study

Selection via IMDB ratings has been previously reported in studies involving VOD services;^11^ however, this method of sample selection has limitations in that we are unable to know whether the highly rated films were widely viewed and we are unable to use audience viewing figures to measure exposure to alcohol, tobacco and HFSS content from these films. However, it is known that VOD programmes may be much easier to access compared with terrestrial television programmes^11^^,^^27^ due to factors such as the ubiquitous nature and general availability of internet^28^ and that ~80% of children aged 5–15 watch some form of VOD content.^30^ Therefore, it is likely original films on VOD services may be a potential source of alcohol, tobacco and HFSS content, which is likely to contribute to youth uptake and consumption. The scope of this study is limited as it only covers services provided by Amazon Prime Instant Video and Netflix watched in the UK for a period of only 1 year, but these services are also reached to population worldwide, and our results apply much more broadly than in the UK.

## Conclusion

VOD films are a potential source of exposure to tobacco, alcohol and HFSS content, which likely leads to increased use and uptake among adolescents. The current regulations are not sufficient to prevent exposure to this content.

## Funding

Saudi Electronic University, the Medical Research Council [grant number MR/K023195/1]; Cancer Research UK [C63710/A27908].

## Notes

**Khaldoon Alfayad,** PhD student

**Rachael L. Murray,** Professor of Health Policy

**John Britton,** Emeritus Professor of Epidemiology

**Alexander B. Barker,** Research Fellow
